# Hapten-Based Cancer Immunotherapy: From Immune Activation to Antitumor Activity

**DOI:** 10.3390/cells15090741

**Published:** 2026-04-22

**Authors:** Iseulys Richert, Lionel Chalus, Benoit Pinteur, Paul Bravetti, Corinne Tortorelli, George Alzeeb, François Ghiringhelli

**Affiliations:** 1 Brenus-Pharma, 69002 Lyon, France; irichert@brenus-pharma.com (I.R.);; 2 University of Bourgogne Franche-Comté, 21000 Dijon, France; 3 Department of Medical Oncology, Centre Georges-François Leclerc, 21000 Dijon, France; 4 Centre de Recherche INSERM LNC-UMR1231, 21000 Dijon, France

**Keywords:** haptenation, immunotherapies, tumor-associated antigens, dendritic cells, delayed-type hypersensitivity

## Abstract

**Highlights:**

**What are the main findings?**

- Haptenation reprograms immune-silent tumors by generating neoepitopes and enhancing antigen presentation, leading to robust CD4^+^ and CD8^+^ T-cell activation and durable antitumor immunity.
- Preclinical and clinical evidence demonstrate that hapten-based cancer immunotherapies induce strong delayed-type hypersensitivity (DTH) responses, clonal T-cell expansion, and tumor regression, while being well tolerated with generally favorable safety profile, with mostly local reactions and rare systemic toxicity.

**What are the implications of the main findings?**

- Hapten-based immunotherapies represent a promising strategy to overcome immune resistance in “cold” tumors and improve responses to immune checkpoint inhibitors.
- Hapten-based immunotherapies support the development of next-generation cancer immunotherapies, including personalized autologous and off-the-shelf allogeneic platforms with broad therapeutic applicability.

**Abstract:**

Hapten-based immunotherapies represent a promising strategy to enhance the immunogenicity of tumor antigens and promote antitumor immune responses. Chemical conjugation of small haptens to antigens generates novel antigenic determinants that increase immune recognition. Mechanistic studies indicate that haptenation enhances antigen uptake, dendritic cell maturation, and the activation of both cellular and humoral immunity. In preclinical models, hapten-modified antigens induce robust immune activation, tumor regression, and durable immune memory. Clinically, dinitrophenyl-modified autologous tumor cell vaccines elicit delayed-type hypersensitivity responses and clonal T-cell expansion, with evidence of clinical activity and a favorable safety profile. However, their clinical benefit remains to be confirmed in larger, randomized studies. Emerging strategies include in situ haptenation and bihaptenized or stressed hapten-modified allogeneic platforms, which aim to expand epitope diversity and enhance immune priming. Hapten-based immunotherapies offer a clinically feasible approach to converting poorly immunogenic tumors into effective immune targets.

## 1. Introduction

Cancer immunotherapy has reshaped oncology by leveraging the immune system’s ability to recognize and eliminate tumor cells. Among the different strategies explored, tumor antigen-based immunotherapies [1,2] aim to stimulate adaptive immune responses against tumor-associated antigens (TAAs) and tumor-specific antigens (TSAs). However, a major challenge lies in the intrinsically low immunogenicity of many tumor antigens, which limits their ability to restore immune surveillance and generate robust cytotoxic T lymphocyte (CTL) responses. Consequently, conventional tumor antigen-based immunotherapies, including peptide-based, dendritic cell (DC)-based, and viral vector vaccines, often fail to generate strong and sustained antitumor immunity in clinical settings, despite promising preclinical results. To address these limitations, researchers have leveraged haptenation, a classical immunological approach that enhances antigen immunogenicity through chemical modification. Haptens are low-molecular-weight chemicals that cannot elicit an immune response on their own but acquire immunogenicity once covalently bound to a larger carrier molecule, typically an endogenous or exogenous protein [3,4]. Crucially, haptenation operates through two sequential and mechanistically distinct processes: first, the de novo generation of novel antigenic sites, and second, the amplification of the immune response through pre-existing hapten-specific immunity. Understanding this sequence is essential to appreciating how hapten-based strategies restore immune surveillance against tumors. Many haptens are reactive electrophilic chemicals or are metabolized into reactive intermediates. These intermediates form covalent bonds with nucleophilic sites, such as NH_2_ groups, on carrier proteins through substitution reactions [4,5]. This chemical conjugation is the first and essential step of haptenation: it physically generates novel antigenic determinants, or neo-epitopes, that are absent from the unmodified protein [5,6]. These newly created epitopes render otherwise immunologically silent antigens visible to the adaptive immune system, enabling T- and B-cell recognition that would not otherwise occur [7,8]. Once novel antigenic sites are established, the immune response can be further boosted by leveraging pre-existing hapten-specific immunity. Individuals previously sensitized to a given hapten harbor memory B and T cells primed against hapten-conjugated epitopes [9,10]. When tumor-associated antigens are haptenated with such molecules, this pre-existing immunity accelerates and amplifies the antitumor response, not by creating new recognition targets, but by recruiting an already-primed immune repertoire against newly hapten-modified tumor antigens [10,11]. Together, these two sequential mechanisms—de novo epitope generation followed by immune amplification—form the immunological basis of hapten-based cancer immunotherapy. Both mechanisms are exploited through two main therapeutic strategies: (i) direct modification of tumor or immune cells, or (ii) hapten-carrier platforms. In the latter, haptens are conjugated to proteins (e.g., dinitrophenyl [DNP]–bovine serum albumin [BSA]) or cells (e.g., autologous tumor cell vaccines, allogeneic cell lines such as stimulated tumor cells [STC]-1010). These platforms enable efficient haptenated antigen delivery, and, particularly for allogeneic formulations, scalable manufacturing, offering key advantages for translational oncology [12]. By enhancing both antigenicity and immunogenicity, haptenation facilitates immune recognition of tumor antigens that might otherwise evade detection, thereby activating both humoral and cellular immune responses. This review examines the immunological rationale, therapeutic potential, and clinical translation of hapten-based immunotherapies. We describe how haptenated antigens enhance antigen presentation, promote DC maturation, and stimulate T- and B-cell responses to counteract tumor-induced immune suppression. Finally, we discuss mechanistic insights from preclinical and clinical studies, highlighting emerging strategies and key challenges shaping the next generation of hapten-based cancer immunotherapies.

## 2. Hapten-Based Immunotherapies: Historical Perspective

The use of small, chemically reactive molecules, known as haptens, to modulate immune responses dates to the early 20th century and has significantly influenced our understanding of immunogenicity, antigen specificity, and tumor immunity. Karl Landsteiner’s landmark 1936 study [13] demonstrated that proteins chemically coupled with diazonium compounds to form azoproteins could elicit specific antibody responses in rabbits. These findings challenged the long-standing belief that only proteins were inherently immunogenic.

Building on these findings, Weigle (1965) [14] demonstrated that chemical conjugation of proteins with compounds such as arsanilic or sulfanilic acid could render them highly immunogenic. This was observed even in contexts where the corresponding native proteins failed to elicit an immune response. For example, animals previously unresponsive to native BSA developed strong antibody responses following exposure to its hapten-conjugated form. Similarly, rabbits immunized with hapten-conjugated thyroglobulin produced antibodies against the antigen. These observations illustrate how subtle structural changes can modulate the way antigens are recognized and processed by the immune system.

Beyond humoral immunity, haptens also influence T-cell responses. In 1974, Shearer [15] demonstrated that trinitrophenyl (TNP)-conjugated cells could stimulate cytotoxic T lymphocytes when presented by genetically matched antigen-presenting cells. This established that haptens introduce novel antigenic determinants recognizable by T cells. These responses were specific and major histocompatibility complex (MHC)-restricted, reflecting the interplay between chemical conjugation and antigen presentation.

T-cell involvement in hapten-induced immunity was later solidified by Tarcic et al. [16] in 1989, who demonstrated that TNP-modified syngeneic spleen cells could stimulate DTH. These responses depended on recognition of hapten-induced molecular alterations rather than the hapten itself and were associated with a small peptide fragment derived from the H-2Dd MHC heavy chain. These findings showed that haptenation can expose previously hidden structural elements, reshaping antigen presentation to promote T-cell activation.

Collectively, these studies, spanning over five decades, illustrate the power of haptenation as an immunomodulatory strategy. These foundational insights established the basis for modern applications of haptenation in cancer immunotherapy [12], including enhancing antigen processing and presentation, promoting DC activation, and stimulating potent tumor-specific immune responses.

## 3. Mechanisms of Hapten-Mediated Immune Activation

**Figure 1:**
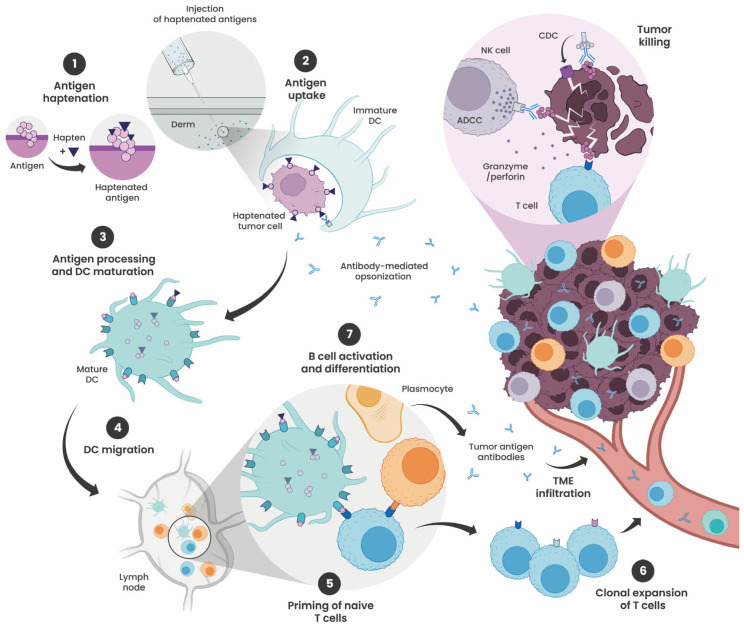
Immune mechanisms triggered by injection of haptenated antigens in non-immunogenic tumors. (1) Haptens are covalently conjugated to tumor antigens, generating hapten-modified neoepitopes on the tumor cell surface. (2) Following intradermal injection, haptenated tumor cells are phagocytosed by immature dendritic cells (DCs) (see Figure 2). (3) Haptenation reprograms antigen processing, generating hapten-modified peptides presented by DCs on MHC class I and II molecules (see Figure 3). Haptenated tumor cells also release danger-associated molecular patterns (DAMPs), including HMGB1 and calreticulin, promoting DC maturation and upregulation of the co-stimulatory molecules CD80 and CD86 (see Figure 4). (4) Mature DCs migrate to the draining lymph nodes, where (5) they present hapten-modified peptides to naïve T cells via MHC–TCR interactions, providing co-stimulatory and cytokine signals that drive T-cell activation (see Figure 5). (6) Activated CD8^+^ T cells undergo clonal expansion and infiltrate the tumor microenvironment (TME), where they mediate direct tumor cell killing. (7) Helper T cells promote B cell activation and differentiation into plasma cells, leading to the secretion of tumor-specific antibodies. These antibodies bind tumor cell antigens and mediate tumor cell elimination through Antibody-Dependent Cellular Cytotoxicity (ADCC) or Complement-Dependent Cytotoxicity (CDC) (see Figure 6). Antibody-mediated opsonization additionally enhances uptake of haptenated tumor cells by antigen-presenting cells, creating a positive feedback loop that further amplifies the immune response.

The limited efficacy of many immunotherapies largely stems from defects in the cancer–immunity cycle, most notably insufficient tumor immunogenicity. Hapten-based therapies can restore antitumor immunity by enhancing immunogenicity at multiple stages of this cycle (Figure 1). Hapten modification of antigens (1) facilitates their uptake by DCs (2), modulates antigen processing to generate novel immunogenic epitopes, and promotes DC maturation (3). After migration (4), these changes amplify T-cell priming (5) and drive robust effector responses (6). In parallel, haptenated antigens present structurally distinct epitopes more readily recognized by B-cell receptors, enhancing B-cell activation and antibody production (7). These tumor-specific antibodies can mediate antibody-dependent cellular cytotoxicity (ADCC) or complement-dependent cytotoxicity (CDC). Through these complementary mechanisms, haptenation reshapes the immune system’s antigen recognition landscape, ultimately improving the therapeutic efficacy of tumor-antigen-based immunotherapies.

Each of these immune events is examined in detail in the sections below, with emphasis on mechanistic and preclinical evidence underpinning haptenation’s role in antitumor immunity.

### 3.1. Antigen Capture and Uptake

**Figure 2:**
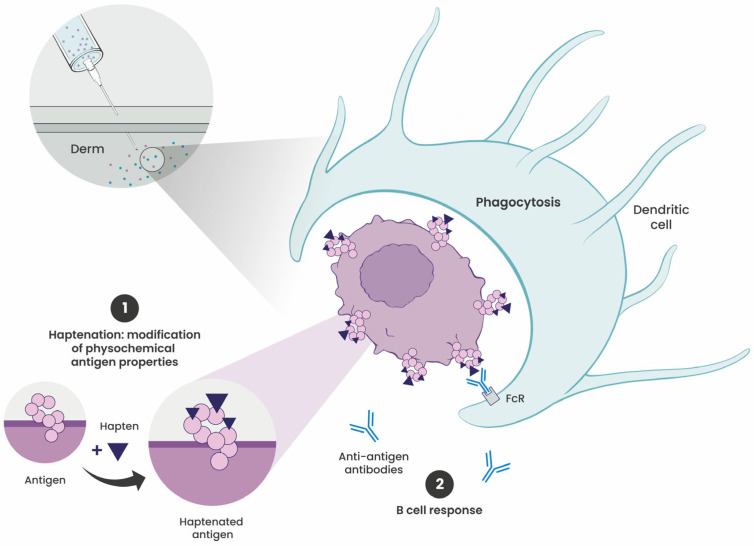
Haptenation enhances antigen uptake by dendritic cells (DCs). (1) Haptenation involves the covalent attachment of haptens to tumor antigens, altering their physicochemical properties—including charge distribution, solubility, and aggregation state. These changes facilitate recognition and uptake of haptenated antigens by DCs. (2) Following prior sensitization to the hapten, anti-hapten antibodies opsonize haptenated tumor cells, promoting Fc receptor (FcR)-dependent uptake by DCs. This amplifies antigen presentation and strengthens downstream T-cell responses.

Figure 2 illustrates the mechanism through which haptenation promotes antigen uptake.

The immunological efficacy of hapten–protein complexes derives from the ability of haptenation to reshape how antigens are captured, processed, and presented by antigen-presenting cells (APCs). Through covalent conjugation, haptens enhance the intrinsic immunogenicity of antigens and alter key biophysical properties, such as charge distribution, solubility, and aggregation state [17]. These structural changes may enhance recognition and uptake by APCs and influence downstream antigen processing. Gefen et al. [17] demonstrated that mannosamine–biotin conjugation alters the isoelectric point of carrier proteins, reflecting major changes in charge and surface chemistry. Confocal microscopy showed that haptenated proteins were efficiently internalized by macrophages and co-localized with lysosomal compartments.

Another key mechanism that enhances antigen uptake is the generation of anti-hapten antibodies after initial exposure. This mechanism will be detailed in Section 3.4. For instance, coating tumor cells with DNP may enable recognition by hapten-specific antibodies, and may promote driving Fc receptor-mediated uptake by DCs. This mechanism potentially boosts DC activation, improves antigen presentation, and strengthens T-cell-mediated antitumor responses in mice [18].

Together, these findings show that haptenation not only alters antigen chemistry and degradation but also creates a structural route to adaptive immune activation that can bypass conventional innate receptor engagement.

### 3.2. Antigen Processing and Presentation

**Figure 3:**
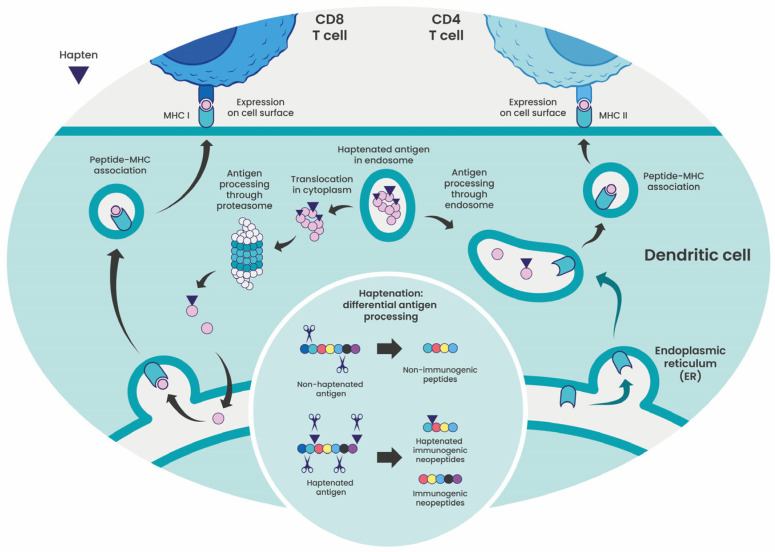
Differential antigen processing of haptenated antigens. After phagocytic uptake of haptenated tumor cells by dendritic cells (DCs), antigens can follow distinct intracellular processing pathways. In the cytosolic pathway, antigens are degraded by the proteasome. The resulting peptides are transported into the endoplasmic reticulum (ER), loaded onto major histocompatibility complex (MHC) class I molecules, and presented on the cell surface to CD8^+^ T cells. Alternatively, in the endosomal pathway, antigens are degraded within late endosomes or lysosomes, loaded onto MHC class II molecules in the endosomal compartment, and presented to CD4^+^ T cells. Compared with their non-haptenated counterparts, haptenated antigens undergo reprogrammed proteolytic cleavage, generating novel immunogenic peptides that may or may not retain the hapten moiety.

Antigen processing and presentation are central to immune surveillance. Endogenous proteins are continuously degraded by the proteasome into short peptides, which are transported into the endoplasmic reticulum, loaded onto MHC class I molecules, and presented on the cell surface for recognition by CD8^+^ T cells. However, this process is limited by the substrate preferences of the proteasome and the transporter associated with antigen processing (TAP) complex, leaving many potential epitopes unproduced or unpresented. Chemical conjugation through haptenation can overcome this bias by reshaping proteolytic processing and peptide–MHC binding. This generates novel haptenated peptides—neoepitopes—that are absent from the native protein (Figure 3).

Proteomic analysis revealed that haptenation modifies proteolytic processing, producing fewer and distinct peptide fragments, some retaining the hapten moiety [17]. Thus, haptenation reprograms protein degradation and peptide presentation pathways, expanding the immunopeptidome.

Haptenation alters both the degradation kinetics of antigens and the repertoire of peptides accessible to MHC molecules, expanding immune visibility of otherwise silent or self-derived proteins. Early insights came from glycoconjugate vaccines [19], in which bacterial polysaccharides are covalently linked to carrier proteins. Upon internalization by APCs, these conjugates are processed within endosomes to generate glycopeptides presented on MHC class II molecules and recognized by carbohydrate-specific CD4^+^ T cells. The efficiency of this process depends on the chemical stability of the glycoconjugate. Although this system primarily involves MHC class II, it illustrates how chemical modification can reveal immunologically silent epitopes by modulating antigen degradation.

Pandey et al. [20] demonstrated that covalent modification of KRAS G12C by the inhibitor sotorasib generates haptenated peptide fragments that are processed and presented on MHC class I molecules. These modified peptides maintain canonical MHC class I binding while introducing a drug-derived chemical moiety that enables specific antibody recognition. The presence of these haptenated complexes was confirmed in mouse tumors. Cryo-EM analysis further demonstrated that haptenation alters antigen processing to yield antibody-recognizable neoepitopes associated with improved antitumor efficacy.

Linette et al. [21] confirmed that related inhibitors, including ARS1620, naturally give rise to haptenated KRAS peptides presented via MHC class I, demonstrating that covalent inhibitor binding can convert intracellular oncogenic protein into immunogenic, tumor-specific antigen. Beyond KRAS, Thomson et al. [22] showed that β-lactam antibiotics such as benzylpenicillin and piperacillin form drug–protein adducts at specific lysine residues, yielding MHC-bound neoepitopes capable of activating both CD4^+^ and CD8^+^ T cells—even in individuals without drug hypersensitivity—highlighting that the position of haptenation critically determines immunogenicity. Building on these mechanistic insights, hapten-based platforms now harness targeted antigen degradation to enhance immune recognition [23]. The targeted antigen degradation-based tumor vaccine (TAgD-TVac) platform, described by Zhao et al., couples tumor-derived proteins to an E3 ligase-recruiting hapten, directing antigens into the ubiquitin–proteasome pathway. This targeted degradation accelerates antigen processing and enhances cross-presentation via MHC class I, eliciting potent CD8^+^ T-cell responses.

Collectively, these findings establish haptenation as a powerful mechanism to reprogram antigen processing and presentation. By chemically modifying proteins, haptenation expands the immunopeptidome, revealing cryptic or novel epitopes that engage both cytotoxic and helper T cells. This dual enhancement of MHC class I and II presentation positions haptenation-based strategies as a promising avenue for next-generation antigen-based therapies capable of overcoming immune tolerance and unveiling previously hidden antigenic landscapes.

### 3.3. Dendritic Cell Maturation to Induce Effective T-Cell Priming

**Figure 4:**
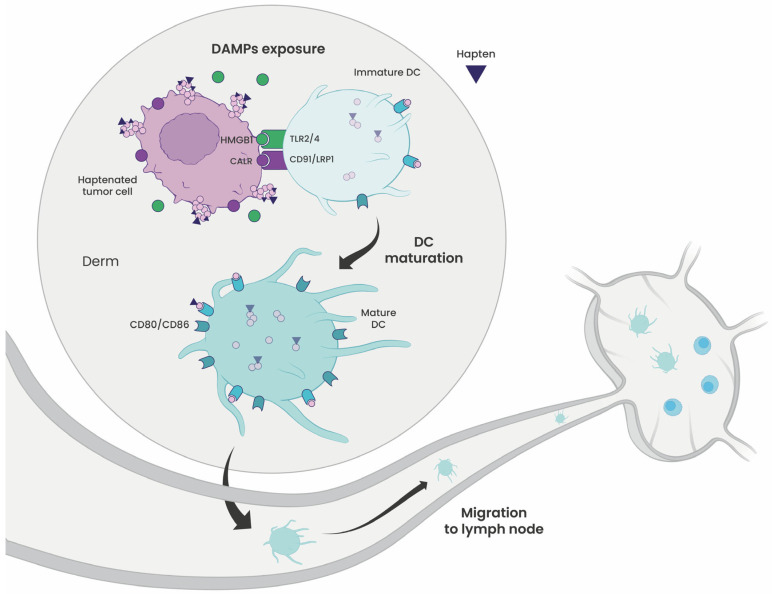
Haptenation promotes dendritic cell (DC) maturation. Haptenation of tumor cells induces the release of damage-associated molecular patterns (DAMPs), including calreticulin (CALR) and high mobility group box 1 (HMGB1). CALR engages CD91/low-density lipoprotein receptor-related protein 1 (LRP1), while HMGB1 signals through Toll-like receptors 2 and 4 (TLR2/4) on immature DCs, triggering DC maturation. This maturation is marked by upregulation of the co-stimulatory molecules CD80 and CD86. Mature DCs subsequently migrate to lymph nodes to prime T-cell responses.

In parallel with reprogramming antigen processing and presentation, haptenation of tumor cells triggers the release of danger signals (damage-associated molecular patterns [DAMPs]) that activate immature DCs (iDCs) (Figure 4). DAMPs such as HMGB1 and calreticulin upregulate the expression of co-stimulatory molecules (CD80, CD86, CD83) and MHC class II in DC [24,25,26].

Experimental models demonstrate that hapten exposure directly drives DC maturation. Rougier et al. [27] showed that Langerhans-like DCs (LLDCs) derived from CD34^+^ progenitors matured upon exposure to strong haptens such as Bandrowski’s base (BB) or fluorescein isothiocyanate (FITC), acquiring an immunostimulatory (CD83^+^ CD86^+^ HLA-DR^++^) phenotype capable of priming autologous naïve T cells. Similarly, Guironnet et al. [28] reported comparable effects with TNP-modified antigens in monocyte-derived DCs. In vitro, iDCs that internalized DNP-modified human serum albumin (HSA) elicited strong primary CD4^+^ T-cell responses, demonstrating that hapten-derived neoepitopes can trigger adaptive immunity even in the absence of inflammation [29].

**Figure 5:**
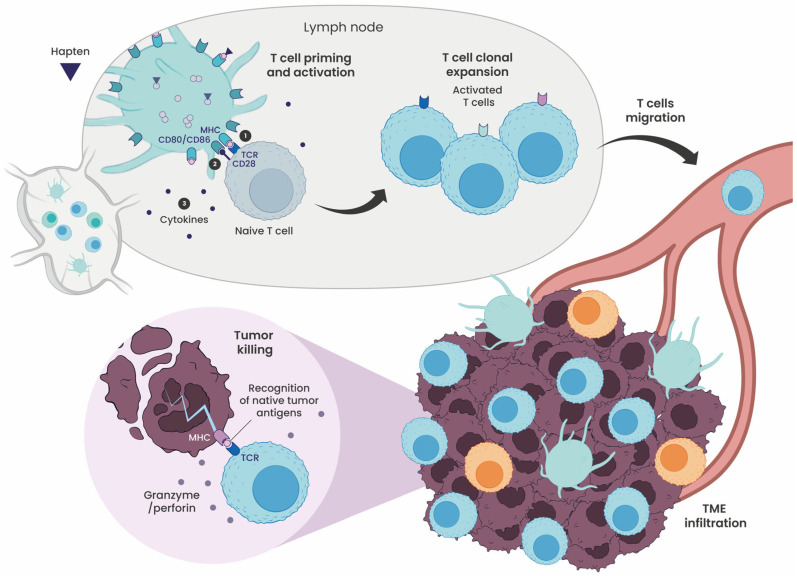
Haptenation drives antigen-specific T-cell expansion, resulting in T-cell-mediated tumor killing. In the lymph nodes, mature dendritic cells (DCs) present antigenic peptides on major histocompatibility complex (MHC) molecules, which are recognized by the T-cell receptor (TCR) on naïve T cells (signal 1). Co-stimulation via CD80/CD86 binding to CD28 (signal 2) and cytokine secretion (signal 3) accompanies this interaction, collectively driving T-cell activation. Activated T cells undergo clonal expansion before migrating to the tumor microenvironment (TME). Within the TME, effector T cells recognize antigen-presenting tumor cells and induce cytotoxic killing through the release of perforin and granzymes, resulting in tumor cell death.

Haptenation improves antigen processing and presentation, increasing the diversity and density of peptide–MHC complexes (signal 1) and strengthening co-stimulatory signaling (signal 2), thereby preventing T-cell anergy and promoting robust CD4^+^ and CD8^+^ T-cell activation (Figure 5). By generating chemically distinct epitopes, haptenation expands the T-cell receptor (TCR) repertoire, recruiting previously unresponsive clones and broadening antigen-specific immunity. Structural studies indicate that these modifications alter TCR–MHC interactions without abolishing antigen recognition, thereby preserving class I-restricted specificity. During the priming phase, T cells activated by hapten peptide complexes rapidly expand and differentiate into effector and memory subsets, forming a durable pool of tumor-reactive lymphocytes.

Bechara et al. [30] proposed that pre-existing reactive naïve TCRs may explain the responsiveness to hapten-conjugated peptides even without prior antigen exposure. Gagnon et al. [31] showed that conserved MHC “hotspots” can preserve TCR–MHC geometry despite peptide modification, indicating that hapten-specific TCRs can maintain HLA class I-restricted recognition. Following priming by hapten-matured DCs, CD8^+^ CTLs mediate tumor cell lysis via perforin and granzyme and Fas–FasL interactions. In parallel, CD4^+^ helper T cells amplify responses through interleukin 2 (IL-2), interferon (IFN)-γ, and tumor necrosis factor (TNF)-α secretion. These cytokines reinforce DC licensing via CD40–CD40L signaling, promoting sustained antigen presentation and CTL function. Functional studies confirm that T-cell subsets recognize haptenated peptides through classical MHC class I and class II pathways, establishing the cooperative basis of hapten-driven immunity [27,29,32,33].

In murine models, TNP-modified syngeneic cells elicit strong T-cell-mediated DTH responses, characterized by local inflammation and lymph node proliferation. These responses are transferable with T cells, suppressed by regulatory lymphocytes in normal mice, and restored under immunodeficient conditions. This demonstrates that haptenation can break peripheral tolerance and activate autoreactive T cells against modified self-antigens [16,34].

These mechanisms illustrate how haptenation drives DC maturation, robust T-cell priming, and TCR repertoire diversification, transforming weakly immunogenic tumor antigens into potent activators of adaptive immunity through coordinated DC–T-cell crosstalk and durable effector programming. These mechanisms likely contribute to enhanced immune activation observed in clinical studies using hapten-based immunotherapies [35,36].

### 3.4. B-Cell Activation and Antibody Production

**Figure 6:**
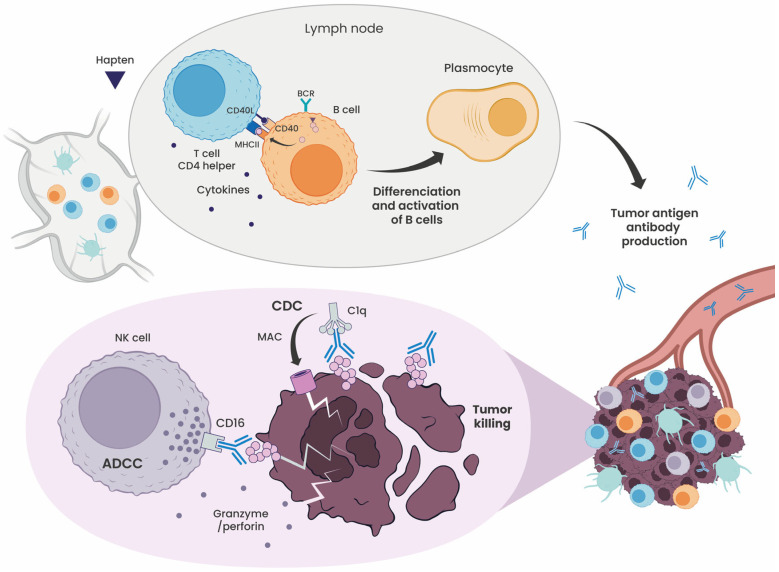
Haptenation drives B-cell activation and antitumor antibody production. Haptenated antigens bind to the B-cell receptor (BCR), triggering antigen internalization. The internalized antigen is processed and presented as peptides on MHC class II molecules to CD4^+^ helper T cells. This interaction drives B-cell activation and differentiation into antibody-secreting plasma cells. Tumor-specific antibodies are secreted into circulation, reach the tumor site, bind tumor antigens, and mediate tumor cell killing via Fc-dependent mechanisms. In antibody-dependent cellular cytotoxicity (ADCC), natural killer (NK) cells are recruited through Fc receptor engagement and lyse opsonized tumor cells. In complement-dependent cytotoxicity (CDC), C1q binds the antibody–antigen complex, activating the complement cascade and triggering membrane attack complex (MAC) formation and tumor cell lysis.

Haptenation also plays a critical role in activating B cells and driving antibody-mediated tumor clearance (Figure 6). Chemical modification of antigens with haptens alters their physicochemical properties, increasing immune visibility and promoting robust polyclonal B-cell responses that engage multiple effector pathways to improve tumor control [18].

The humoral response begins when B cells recognize hapten-conjugated tumor antigens via their B-cell receptors (BCRs). Upon binding, B cells internalize the antigen, process it, and present hapten-modified peptides on MHC class II molecules to CD4^+^ T-helper cells. These T cells provide critical co-stimulatory signals, through CD40L engagement and cytokines such as IL-4 and IL-21, driving B-cell proliferation, immunoglobulin class switching, and differentiation into plasma cells. This interaction supports germinal center formation, where B cells undergo affinity maturation and generate long-lived memory B cells. The resulting antibodies target both the hapten and surrounding tumor epitopes, enabling broad and synergistic tumor targeting. These antibodies mediate tumor destruction through multiple established mechanisms. In ADCC, Fc receptor-bearing natural killer (NK) cells recognize the Fc portion of anti-TAA antibodies and lyse opsonized tumor cells. In complement-dependent cytotoxicity (CDC), binding of antibody-coated tumor cells activates the classical complement pathway, resulting in membrane attack complex (MAC) formation and lysis. Antibody-dependent cellular phagocytosis (ADCP) enables macrophages and DCs to internalize antibody-opsonized tumor cells, promoting both direct clearance and enhanced cross-presentation of tumor antigens. Collectively, these mechanisms not only eliminate tumors but also amplify downstream T-cell responses [37,38,39,40].

In Galili’s α-gal model [41], α-gal epitopes are experimentally introduced to tumor cells as a form of haptenation to harness abundant natural anti-Gal antibodies. Purified α-gal glycolipids are administered as micelles and spontaneously insert into tumor cell membranes, displaying α-gal epitopes on the cell surface. These hapten-like structures are rapidly recognized by circulating anti-Gal IgM and IgG. This interaction activates the complement cascade and triggers the release of chemotactic factors C5a and C3a, which recruit DCs and macrophages to the tumor site. These APCs internalize anti-Gal–opsonized tumor cells via Fc/Fc-gamma receptors (FcγR)-mediated uptake, process TAAs, and present them on MHC class I and II molecules. The resulting activation of tumor-specific CD8^+^ and CD4^+^ T cells generates a potent, systemic immune response that eliminates local and distant tumor cells without inducing autoimmunity.

In a complementary approach, Schrand et al. [18] introduced a two-step haptenation strategy: mice were first immunized with DNP–KLH to generate anti-DNP antibodies, then treated with aptamer–DNP conjugates targeting tumor ligands. This recruited pre-existing antibodies to tumors, producing CD4^+^ T-cell- and B-cell-dependent antitumor responses, while CD8^+^ T cells were dispensable. The recruited antibodies mediated ADCC and enhanced uptake by FcR^+^ APCs, further reinforcing adaptive immunity. Yu et al. demonstrated that hapten modification of tumor-associated antigens (TAAs) can enhance tumor immunogenicity and induce the generation of tumor-specific antibodies [42]. Using Hapten-Enhanced Local Chemotherapy (HELC), the authors showed that haptens chemically modify endogenous TAAs to form neo-TAAs, which elicit robust humoral immune responses. This approach leads to the production of in situ tumor-associated autoantibodies (iTAAs) targeting oncogenic nuclear antigens such as survivin, c-MYC, and p53. The resulting tumor-specific antibodies mediate complement activation and contribute to effective tumor cell targeting, supporting haptenation as a strategy to increase tumor immunogenicity.

Collectively, these studies show that hapten-based immunotherapies not only restore antigen immunogenicity but also engage B cells and pre-existing antibodies to orchestrate a multifaceted humoral response that synergizes with cellular immunity for durable tumor control.

## 4. Translational Evidence and Clinical Applications

Hapten-based immunotherapies translate mechanistic principles into clinical practice by enhancing the immunogenicity of poorly recognized tumor antigens. This is achieved through neoepitope generation, improved antigen uptake and presentation, and modulation of antigen-processing pathways—collectively activating cytotoxic and helper T cells as well as B cell-mediated antibody responses. Preclinical and early clinical studies have confirmed that haptenation can overcome immune tolerance, remodel the tumor–immune interface, and induce durable immune memory. Strategies range from ex vivo modification of autologous or allogeneic tumor cells to in situ haptenation and hapten–peptide conjugates, each offering distinct advantages in antigenic breadth, delivery feasibility, and immune potency.

### 4.1. Preclinical Evidence for Hapten-Based Immunotherapies

The preclinical foundation for hapten-based immunotherapies is illustrated by a series of mechanistic and translational studies (Table 1).

Fujiwara et al. [43] demonstrated that in situ haptenation can transform tumors into immunogenic targets. Using a syngeneic X5563 plasmacytoma model in C3H/HeN mice, they showed that intratumoral injection of trinitrochlorobenzene (TNCB) generated TNP-modified tumor cells capable of eliciting strong helper T-cell responses. Haptenation alone was insufficient; however, when preceded by suppressor T-cell elimination using either TNP-D-GL or CY (each independently effective), it produced high rates of complete tumor regression. More than 90% of mice with regressed tumors resisted rechallenge, confirming the induction of durable, tumor-specific immunity. This work established amplified helper T cells as key mediators of CTL activation and provided early evidence that haptenation can reprogram the tumor milieu to favor systemic immune protection.

Sato et al. [10] extended this concept to human melanoma. Using autologous melanoma cells modified with DNP, they found that T cells specifically recognized a single dominant peptide fraction derived from DNP-modified cells, inducing strong IFN-γ secretion. This response was MHC class I-restricted and strictly dependent on DNP conjugation, with no reactivity to unmodified peptides. Mass spectrometry confirmed DNP incorporation exclusively in the immunogenic fraction, providing molecular evidence that haptenation generates novel MHC-presented tumor peptides capable of eliciting potent human T-cell responses.

Sojka et al. [44] evaluated DNP-haptenated autologous tumor cell (ATC) vaccines in a syngeneic post-surgical breast cancer model. Vaccines were administered after low-dose cyclophosphamide (CY) and combined with Bacillus Calmette–Guérin (BCG). This combination significantly improved relapse-free survival (RFS) compared with the unmodified ATC vaccine or saline controls. Depletion of CD4^+^ or CD8^+^ T cells or neutralization of IFN-γ or TNF abolished efficacy, indicating a Th1-biased, T cell-dependent mechanism. Together, these findings showed that durable tumor control in the post-surgical setting depends on the synergy between hapten-induced immunogenicity, CY-mediated immune priming, and pro-inflammatory cytokine signaling.

This concept was further advanced by Alzeeb et al. [45] through the development of the STC platform for colorectal cancer (CRC). This allogeneic, off-the-shelf immunotherapy combines physical and chemical tumor cell stress with DNFB haptenation to broaden antigen immunogenicity. They developed murine surrogate and assessed their antitumor immune activity in vivo. In the CT26 model, the allogeneic three-cell-line formulation (3CL-SH) combined with an immunostimulant regimen (low-dose CY and granulocyte-macrophage colony-stimulating factor (GM-CSF)) significantly delayed tumor growth and improved overall survival compared with controls, consistent with its broader antigenic repertoire confirmed by proteomic analysis. Notably, 3CL-SH remained effective in the anti–programmed cell death-1 (PD-1)–resistant MC38 model, prolonging survival and increasing intratumoral CD8^+^ T cells and M1 macrophage infiltration. The treatment was well tolerated.

To facilitate translation toward human systems, Alzeeb et al. [46] further evaluated the activity of the human version of STC-1010 in an ex vivo immunological assay using monocyte-derived dendritic cells (mDCs). Preliminary findings from a conference abstract suggest that STC-1010 haptenated peptides are presented by mature DC, which show increased IL-8 and IL-12 secretion and reduced IL-10 levels. These activated DCs primed autologous CD8^+^ T cells to promote apoptosis in CRC cells with consistent reproducibility across four independent production batches. These findings suggest translational potential but warrant peer-reviewed confirmation.

Taken together, these preclinical studies support the concept that haptenation can enhance tumor immunogenicity and antitumor immune responses in preclinical settings.

### 4.2. Clinical Trials and Emerging Therapeutic Applications

Clinical investigations of hapten-based immunotherapies have provided critical insights into overcoming tumor-induced immune tolerance and enhancing tumor immunogenicity (Table 2). Detailed study-level characteristics and results are provided in Supplementary Table S1.

A key biomarker of cellular immunity in hapten-based immunotherapy trials is the DTH response, assessed by intradermal injection of hapten–carrier conjugates and measurement of localized skin inflammation as a readout of functional T-cell activity.

The pioneering work of Berd and colleagues established the mechanistic and clinical rationale for hapten-based immunotherapy. The 1986 study [58] first demonstrated that pretreatment with low-dose CY enhanced immune priming and induced durable tumor regressions in patients with metastatic melanoma, outcomes not observed with the vaccine alone. In follow-up studies, autologous melanoma cells were haptenated with DNFB and administered following topical DNP sensitization and low-dose CY. The treatment induced visible tumor-site inflammation in 14 of 24 patients, with histologic evidence of CD8^+^ and HLA-DR^+^ T-cell infiltration with satellitosis and necrosis [47]. Post-treatment lesions contained activated CD8^+^ tumor-infiltrating lymphocytes (TILs) co-expressing HLA-DR, CD69, and ganglioside GD3, though with minimal IL-2 receptor expression, suggesting activation without high proliferative capacity [9]. Manne et al. [36] further reported clonal intratumoral T-cell expansions in 9 of 10 patients post-vaccination. These expansions were absent in peripheral blood, predominantly involved CD8^+^ cells, and coincided with local inflammation and partial regression in some lesions, suggesting selective in situ immune activation. Collectively, these results demonstrated that haptenation could remodel the tumor microenvironment (TME), converting “immune-cold” tumors into active immune targets.

Further clinical studies confirmed the immunogenicity, safety, and prognostic value of DNP-modified autologous melanoma vaccines. In larger cohorts of patients with stage III (adjuvant) and stage IV (metastatic) disease, 95% developed a strong DTH response to haptenated tumor cells, and approximately half also reacted to unmodified autologous cells [50]. In patients with resected stage III melanoma, the five-year OS was 44%, with improved outcomes in those who mounted DTH responses to unmodified autologous tumor cells (5-year OS 59.3% vs. 29.3%) [49]. The DTH response was unrelated to the number of live tumor cells administered per dose; however, vaccines containing ≤50% live cells produced higher DTH rates than those with >50%, underscoring that dead tumor cells may contribute to immunogenicity [50]. Regarding tolerability, the treatment was consistently well tolerated across studies. Local injection-site reactions were universal but mild, and their intensity decreased with BCG dose reduction, suggesting they are at least partly adjuvant-driven. Systemic reactions were rare (<5% of patients reported fever or chills), with no significant changes in blood counts, no evidence of autoimmunity, and no cases of vitiligo [49,50].

Mechanistic studies further clarified how haptenation shapes immune activation. Sato et al. (1995) [51] demonstrated that DNP-haptenated autologous melanoma vaccines induce hapten-specific, MHC class I-restricted cytotoxic responses. Expanded CD8^+^ T cells were the primary effectors against DNP-modified autologous melanoma cells, producing IFN-γ but not IL-4, consistent with a Th1 profile. Both CD4^+^ and CD8^+^ subpopulations responded to haptenated stimulators, though cytolytic activity against tumor targets was restricted to the CD8^+^ subpopulation.

Lotem and colleagues [52,53,54,55] confirmed and extended these findings in a series of phase II trials. In adjuvant settings, strong DTH responses predicted prolonged disease-free survival (DFS) and OS, with multivariate analyses confirming DTH as an independent prognostic marker [53,54]. Mechanistic studies revealed that vaccine-induced CD4^+^ and CD8^+^ responses required MHC class II expression and were associated with anti-livin antibody formation [55]. High expression of clustered cancer-testis antigens (CTAG2, MAGEA1, SSX1, SSX4) correlated with improved survival in vaccinated patients but not in non-vaccinated cohort [53]. This association suggests that hapten-based vaccination may unmask immune recognition of CTA-related tumor antigens. Patients previously vaccinated also showed improved responses to ipilimumab, indicating potential for durable immune memory and therapeutic synergy with checkpoint inhibition [53]. IL-2 co-administration was evaluated in advanced melanoma [52]. Combined therapy increased objective response rates (ORR) to ~35%, with complete and partial regressions and a median OS of 40 months [52]. Safety across the Lotem studies was favorable, with no significant systemic toxicity and no grade 3–4 adverse events; local injection-site reactions resolved, leaving atrophic scars [54]. Two patients who experienced tumor regression developed vitiligo in areas of regressing metastases, consistent with melanocyte-directed immune activation; whether these patients also received IL-2 co-administration was not reported [52].

Expanding the application of hapten-based immunotherapies beyond melanoma, Bota et al. [56,57] conducted clinical studies in patients with recurrent glioblastoma (rGBM). The multivalent vaccine ERC1671 (Gliovac™)—combining autologous and allogeneic haptenated tumor antigens—produced a median OS of 12.1 months compared with 7.6 months in controls [56]. Peripheral CD4^+^ T-cell counts correlated strongly with OS, underscoring the role of helper T-cell activity in therapeutic efficacy. A subsequent cohort study of SITOIGANAP reported a median OS of 19.6 months overall, extending to 30.6 months in patients completing ≥6 cycles [57]. Both regimens were well tolerated, with no treatment-related serious adverse events, no grade 4–5 toxicities, and only mild injection-site reactions and transient systemic symptoms [56,57].

The ongoing first-in-human, Phase I/IIa BreAK CRC-001 trial (NCT06934538 [59]) is evaluating STC-1010 combined with low-dose CY and GM-CSF immunostimulants and standard chemotherapy (mFOLFOX6 ± bevacizumab) in metastatic MSS CRC patients. Exploratory endpoints include DTH response and immune biomarker profiling, which will provide direct mechanistic evidence of haptenation’s contribution in this setting.

Several important limitations in these clinical trials must be acknowledged. Most trials were small, single-arm, and lacked randomized placebo-controlled designs, making it difficult to isolate the contribution of haptenation from that of co-administered adjuvants such as BCG, CY, or IL-2. Several publications from the Berd group report analyses of overlapping patient cohorts within a single clinical development program; patient numbers across individual studies should therefore not be considered additive.

The mechanistic link between haptenation specifically and clinical benefit has rarely been formally evaluated against non-haptenated vaccine controls in the same patient population. Addressing these limitations through adequately powered, randomized trials will be essential to establish the definitive clinical antitumor efficiency of hapten-based immunotherapies. Accordingly, these studies should be interpreted as hypothesis-generating rather than practice-changing clinical evidence.

Collectively, clinical data across melanoma and glioblastoma show that haptenation promotes Th1-polarized cytotoxic T-cell responses, with favorable safety profiles across studies. Predictive biomarkers, including DTH positivity, CTA expression, and CD4^+^ T-cell expansion, correlate with improved survival. By reshaping the tumor-immune interface, hapten-based immunotherapies are a versatile and promising platform especially in tumors with low baseline immunogenicity.

## 5. Challenges and Future Directions in Hapten-Based Cancer Immunotherapy

The use of hapten-based immunotherapies has emerged as a promising strategy to enhance immune recognition of tumor cells and elicit robust, antigen-specific immune responses. This approach has shown encouraging preclinical and early clinical success, particularly in melanoma and glioblastoma. However, translation into clinical practice remains challenging due to patient-specific variability, complex manufacturing processes, potential safety concerns, and difficulties in standardizing delivery. Addressing these limitations is essential to realize the full therapeutic potential of this strategy.

A major challenge lies in the individualized production of hapten-based therapies, particularly when using autologous tumor cells. These processes require patient-specific tumor procurement, ex vivo haptenation, and rigorous quality control, resulting in scalability barriers, high manufacturing costs, regulatory complexity, and potential delays. Moreover, autologous vaccines may not capture the full tumor heterogeneity, leading to incomplete immune targeting. Allogeneic, off-the-shelf platforms aim to overcome these limitations by providing broader antigenic coverage and standardized manufacturing. Regarding delivery, intratumoral injection of hapten-conjugated agents has been explored as an emerging strategy across multiple cancer types. In this approach—termed hapten-enhanced cytotoxic drug intratumoral injection (HECDI) or ultra-minimum incision personalized intratumoral chemoimmunotherapy (UMIPIC)—a hapten such as penicillin is combined with cytotoxic drugs and injected directly into the tumor. This engages the autologous coagulum as a natural antigen delivery matrix and triggers local immune cell activation. Clinical studies in advanced pancreatic cancer [60], hepatocellular carcinoma [61], and lung cancer [62] have consistently shown that the hapten-containing regimen are associated with longer overall survival in these studies. However, the specific contribution of haptenation cannot yet be definitively separated from other treatment components. Mechanistically, this intratumoral haptenation appears to modify tumor-associated antigens in situ, generating induced tumor-associated autoantibodies (iTAAs) that can target intracellular oncogenic antigens [42]. However, these studies originate from a single research group, with modest sample sizes and limited methodological reporting; independent replication across diverse patient populations will be essential to confirm these findings and establish the clinical utility of intratumoral haptenation as a delivery strategy.

Although hapten-based immunotherapies enhance tumor immunogenicity, their efficacy depends on the patient’s baseline immune function, including intact antigen processing and effective cytotoxic T-cell induction. Therefore, selecting patients with relatively preserved immune competence, such as those receiving first-line therapy, is critical to maximizing clinical benefit.

Some studies have suggested that hapten-based immunotherapies primarily elicit responses against haptenated antigens only, rather than native tumor antigens [10]. However, clinical observations, such as DTH reactions to both haptenated and unmodified tumor cells [48,49,50,52,53,54], demonstrate that these therapies can also induce immune responses against non-haptenated antigens. The post-treatment increase in antibodies targeting TAAs further supports the notion that hapten-based therapies can stimulate broader antitumor immunity beyond hapten-specific responses [42]. Consistently, both preclinical and clinical data demonstrate that hapten-based therapies are capable of mediating cytotoxicity against unmodified tumor cells [9,10,44,47,48,49,52,53].

Safety remains another critical consideration. Free haptens are chemically reactive, low-molecular-weight compounds that can bind to endogenous proteins, potentially triggering allergic or immune-mediated hypersensitivity reactions [63]. However, clinical experience to date suggests that when a hapten is conjugated to tumor cells under controlled conditions, this risk is manageable. Consistent with this, across the hapten-based clinical programs reviewed here, over 300 patients received hapten-based immunotherapies without autoimmune adverse events, suggesting that the ability to distinguish self from non-self is preserved after treatment.

Tumor-mediated immune evasion mechanisms, such as MHC downregulation, antigen loss or downregulation, immunosuppressive microenvironments, and regulatory T-cell activity, can limit the activity of antigen-based immunotherapies [64,65]. Interestingly, some hapten-based immunotherapies, including STC-1010 [45] and other DNP-conjugate models, have shown activity even within immunosuppressive TMEs. Cyclophosphamide exerts Treg-depleting effects that may synergize with hapten-based strategies in immunosuppressive TMEs, and its incorporation into combination regimens warrants further investigation.

Emerging solutions include bihaptenated vaccines that broaden antigenic recognition and reduce single-epitope escape, as well as combination regimens integrating immune checkpoint inhibitors (anti-PD-1, anti-CTLA-4) or cytokine adjuvants (IL-2, GM-CSF, IFN-α). Additional strategies, such as adoptive T-cell transfer and biomaterial-based delivery systems, further enhance antigen presentation, prolong immune activation, and may unlock the full therapeutic potential of hapten-based immunotherapies.

## 6. Conclusions

Hapten-based immunotherapies represent a transformative strategy in immuno-oncology, capable of reprogramming immunologically silent tumors into highly immunogenic targets. By covalently modifying antigens with chemically reactive haptens, this approach enhances antigen uptake, processing, and presentation by DCs and B cells. This facilitates the generation of novel neoepitopes and robust activation of both cellular and humoral immunity. This dual-arm engagement distinguishes haptenation from conventional tumor-antigen-based immunotherapy platforms, which often fail to elicit sustained cytotoxic responses in poorly immunogenic tumors.

Extensive preclinical and clinical studies in melanoma, CRC, and glioblastoma have demonstrated that hapten-based immunotherapies can induce DTH, clonal T-cell expansion, and antibody-mediated effector responses leading to tumor regression, reduced recurrence, and prolonged survival. Recent translational advances include off-the-shelf, multi-antigenic platforms such as STC-1010, personalized autologous DNP-modified tumor cell formulations, and combinatorial regimens that integrate haptenation with checkpoint inhibitors or cytokine adjuvants. These innovations expand the therapeutic scope of hapten-based immunotherapy to immune-cold and checkpoint-refractory tumor settings.

Despite these advances, critical translational challenges remain. The complexity of individualized treatment manufacturing, variability in immune responsiveness, risks of autoimmunity, and potential immune escape through antigen downregulation demand continued refinement. Current research focuses on bihaptenation, intratumoral delivery, off-the-shelf immunotherapy platforms based on stressed and haptenated antigens and biomarker-guided patient stratification using immune profiling.

Looking ahead, hapten-based immunotherapies may unmask cryptic antigenic landscapes, overcome immune tolerance, and synergize with other immunomodulatory therapies underscoring their clinical potential. With continued innovation and rigorous validation, hapten-based immunotherapies may offer renewed hope for patients with immune cold tumors historically resistant to treatment.

## Figures and Tables

**Table 1:** Preclinical studies on hapten-based immunotherapies.

Model	Treatment Type	Outcome Measures	Key Findings	Ref.
X5563 plasmacytoma in C3H/HeN mice Mouse (syngeneic)	In situ haptenation of tumor cells via intratumoral TNCB injection Pre-treatment: TNP-D-GL or CY (to eliminate suppressor T cells) TNCB skin sensitization	Tumor regression CTL and Th cell activity DTH response Resistance to tumor rechallenge	Pretreatment with TNP-D-GL or CY (each independently): amplified hapten-specific Th activity by eliminating suppressor T cells. Intratumoral TNCB injection in pre-treated mice: complete tumor regression in a significantly higher proportion of animals. 90% of mice with regressed tumors: complete resistance to rechallenge	[43]
Autologous melanoma model; EBV-transformed autologous B lymphoblasts as APCs Human (ex vivo)	DNP-modified autologous melanoma cells CY prior to vaccination; BCG as adjuvant	IFN-γ production (functional assay) Mass spectrometric identification of DNP-modified peptides	T cells specifically recognized a single DNP-modified peptide fraction (MHC class I-restricted); no response to unmodified peptides. Mass spectrometry confirmed DNP incorporation exclusively in the immunogenic fraction, identifying hapten-modified MHC-associated peptides as the immunogenic target.	[10]
410.4 murine mammary carcinoma (post-surgical excision model) Mouse (syngeneic)	DNP-modified, irradiated, autologous 410.4 tumor cells (ATC) CY prior to vaccination; BCG as adjuvant	RFS T-cell subset dependency Cytokine dependency (IFN-γ, TNF)	CY + DNP-ATC + BCG significantly improved RFS vs. unmodified ATC or saline control. Both CD4^+^/CD8^+^ T cells were required for therapeutic benefit. IFN-γ and TNF were functionally essential mediators of the anti-metastatic effect	[44]
CT26 colorectal carcinoma; MC38 anti-PD-1 resistant model Mouse (syngeneic)	Allogeneic, haptenated physically stressed tumor cells: 1 cell line (1CL) or 3 cell lines (3CL) Combined with immunostimulants: CY + GM-CSF	Tumor growth OS Proteomic antigen diversity Immune cell infiltration (CD8^+^ T-cells, M1 macrophages)	In CT26, 3CL + IS provided superior tumor control and survival vs. 1CL or control. 3CL vaccine covered a wider range of tumor-related proteins, supporting a multi-specific antitumor immune response. In anti-PD-1-resistant MC38, 3CL + IS improved OS and increased intratumoral CD8^+^ T cell and M1 macrophage. Treatment was well tolerated with no local or systemic toxicity.	[45]
mDCs and CD8^+^ T cells from independent donors; CRC target cell lines Human (ex vivo)	STC-1010: allogeneic, haptenated, multiply stressed CRC cell lines (serum depletion, irradiation, heat-shock, chemotherapy)	DC antigen uptake and haptenized epitope presentation Cytokine secretion Tumor cell apoptosis	STC-1010 induced IL-8 and IL-12 secretion and reduced IL-10 during mDC maturation; mDCs presented haptenized epitopes after co-culture. CD8^+^ T cell primed with STC-1010 treated mDC induce apoptosis of cancer cells Preliminary data suggested batch-to-batch consistency across four production batches	[46] *

* Conference abstract [46]; findings are preliminary and pending peer-reviewed publication. APC: Antigen-presenting cell; ATC: Autologous tumor cells; BCG: Bacillus Calmette-Guérin; CL: Cell line; CRC: Colorectal cancer; CTL: Cytotoxic T lymphocyte; CY: Cyclophosphamide; DC: Dendritic cell; DNP: Dinitrophenyl; DTH: Delayed-type hypersensitivity; EBV: Epstein-Barr virus; GM-CSF: Granulocyte-macrophage colony-stimulating factor; IFN-γ: Interferon-gamma; IL: Interleukin; mDC: Monocyte-derived dendritic cell; MHC: Major histocompatibility complex; OS: Overall survival; RFS: Relapse-free survival; STC: Stimulated tumor cells; Th: Helper T cell; TNCB: Trinitrochlorobenzene; TNF: Tumor necrosis factor; TNP: Trinitrophenyl; TNP-D-GL: Trinitrophenyl-poly-D-glutamic acid-lysine.

**Table 2:** Clinical studies evaluating the efficacy and immune response of hapten-based immunotherapies.

Platform	Study Design and Indication	Main Clinical Outcomes	Safety	Key Findings	Key Ref.
**Hapten-modified autologous melanoma vaccine (M-Vax/DNP vaccine) mixed with BCG**	Phase I/II; single arm; no randomized control Metastatic and resected stage III–IV melanoma	Tumor regression (5/24), disease stabilization (2/24) [47] 11 clinical objective response (2CR, 4PR, 5 mixed) out of 83 Stage IV patients [48] 5-year OS 44% in stage III; 5-year OS 59.3% vs. 29.3% in DTH responders vs. non-responders [49]	Local injection-site reactions (papules/pustules with occasional ulceration) in all patients; intensity reduced with BCG dose reduction Systemic reactions rare (<5%). No blood count changes, no autoimmunity, no vitiligo [49,50]	DTH Positive DTH to haptenated cells in 95% of patients; half also responded to unmodified cells Positive DTH response was associated with improved 5-year OS Vaccines with ≤50% live cells produced higher DTH rates Immunological response Increased TIL infiltration, predominantly CD8^+^ T cells Clonal T cell expansion post-vaccination (9/10 patients) [36] PBL proliferation to DNP-modified autologous cells [51] IFN-γ–producing T cells (5/11 patients), including CD8^+^ cytotoxic cells [51] Anti-DNP antibodies detected in patient serum post-treatment [51]	[36,47,48,49,50,51]
**Hapten-modified autologous melanoma vaccine combined with IL-2, checkpoint blockade (ipilimumab)**	Phase II; single-arm (IL-2 combination [52]); retrospective comparison with non-vaccinated cohort (ipilimumab combination [53]); no randomized placebo control High-risk resected stage III and metastatic stage IV melanoma (adjuvant and therapeutic settings)	ORR 35% (4 CR, 8 PR; median OS 40 months; 10/12 responders also received IL-2 and demonstrated enhanced efficacy [52] 3-year OS 46% in vaccinated vs. 19% in non-vaccinated patients receiving ipilimumab [53]	No grade 3–4 adverse events. No significant systemic toxicity. Local injection-site reactions resolving with atrophic scars [54] Two patients developed vitiligo; IL-2 co-administration status in these patients not reported [52]	DTH Stronger DTH responses were associated with longer OS Immunological response Increased CD8^+^ T-cell responses post-vaccination CD4^+^ T-cell responses correlated with OS in adjuvant group [55] Vaccine-induced responses required MHC class II expression [55] Anti-livin IgG formation associated with prolonged OS [55]	[52,53,54,55]
**Allogeneic/autologous haptenated tumor cell vaccine** **(SITOIGANAP/ERC1671/Gliovac™) combined with bevacizumab ± nivolumab or pembrolizumab**	Phase II, randomized vs. placebo + bevacizumab [56]; prospective single-arm cohort study; no haptenated vs. non-haptenated control arm Recurrent glioblastoma (rGBM)	Median OS 12.1 months (vaccinated) vs. 7.6 months (placebo + bevacizumab) [56] Radiologic responses in 75% of vaccinated patients [56] Median OS 19.6 months overall; 30.6 months in patients completing ≥6 cycles; 1-year survival 90% in ≥6 cycle group [57]	Equal distribution of AEs between active and placebo groups [56] No treatment-related SAEs [57] No grade 4–5 toxicities in either study; mild injection-site reactions (induration, erythema, ulceration) Transient self-limiting fever and chills	Peripheral CD4^+^ T cell counts strongly correlated with OS in treated groups in both studies, underscoring the role of helper T cell activity in therapeutic response No other immune biomarkers reported	[56,57]

BCG: Bacillus Calmette-Guérin; CR: Complete response; DNP: Dinitrophenyl; DTH: Delayed-type hypersensitivity; IFN-γ: Interferon-gamma; IL: Interleukin; MHC: Major histocompatibility complex; ORR: Overall response rate; OS: Overall survival; PBL: Peripheral blood lymphocytes; PR: Partial response; rGBM: Recurrent glioblastoma; SAE: Serious adverse event; TIL: Tumor-infiltrating lymphocytes.

## Data Availability

No new data were created or analyzed in this study. Data sharing does not apply to this article.

## Supplementary Materials

The following supporting information can be downloaded at: https://www.mdpi.com/article/10.3390/cells15090741/s1, Table S1: Clinical studies of hapten-based immunotherapies.

## Abbreviations

The following abbreviations are used in this manuscript:

1CL-SH	Single-cell-line treatment
3CL-SH	Three-cell-line treatment
ADCC	Antibody-dependent cellular cytotoxicity
ADCP	Antibody-dependent cellular phagocytosis
APCs	Antigen-presenting cells
ATC	Autologous tumor cell
BCR	B-cell receptor
BCG	Bacillus Calmette–Guérin
BSA	Bovine serum albumin
CDC	Complement-dependent cytotoxicity
CRC	Colorectal cancer
CTA	Cancer-testis antigen
CTL	Cytotoxic T lymphocyte
CY	Cyclophosphamide
DAMP	Damage-associated molecular patterns
DC	Dendritic cell
DFS	Disease-free survival
DNFB	Dinitrofluorobenzene
DNP	Dinitrophenyl
DTH	Delayed-type hypersensitivity
FcγR	Fc-gamma receptor
FITC	Fluorescein isothiocyanate
GM-CSF	Granulocyte-macrophage colony-stimulating factor
H	Haptenation
HSA	Human serum albumin
HLA	Human leukocyte antigen
iDCs	Immature DCs
IFN	Interferon
IL	Interleukin
LLDCs	Langerhans-like DCs
MAC	Membrane attack complex
MHC	Major histocompatibility complex
moDCs	Monocyte-derived dendritic cells
NK	Natural killer
ORR	Objective response rate
OS	Overall survival
PBMCs	Peripheral blood mononuclear cells
PD-1	Programmed cell death-1
RFS	Relapse-free survival
rGBM	Recurrent glioblastoma
S	Stressed
STC	Stimulated tumor cells
TAA	Tumor-associated antigens
TAgD-TVac	Targeted antigen degradation-based tumor vaccine
TAP	Transporter associated with antigen processing
TCR	T-cell receptor
TILs	Tumor-infiltrating lymphocytes
TME	Tumor microenvironment
TNCB	Trinitrochlorobenzene
TNF	Tumor necrosis factor
TNP	Trinitrophenyl
TSA	Tumor-specific antigen
