# Supplementary material for: Hapten-Based Cancer Immunotherapy: From Immune Activation to Antitumor Activity
Source: Cells. 2026 Apr 22;15(9):741. doi: 10.3390/cells15090741 (PMC13162954; doi:10.3390/cells15090741)
Supplement: Supplementary file 1 [file cells-15-00741-s001.zip › cells-4197651-supplementary-Table S1.pdf]

Number of Patients (n=)	Treatment Groups	Vaccine Composition	Treatment protocol	Treatment Duration	Outcomes Measured	Key Findings	Ref
**Melanoma**							
n=24 patients<br>with metastatic, surgically incurable melanoma	**Single-arm treatment group:** DNP-modified autologous tumor vaccine	Autologous, cryopreserved melanoma cells<br>Irradiated (25 Gy)<br>Haptenated<br>Mixed with BCG	CY (300 mg/m² i.v.) before treatment<br>Topical DNP sensitization<br>Monthly i.d. injections (every 28d)	2–4 months, with vaccine administered every 28d (2–4 injections).	DTH<br>Clinical signs of inflammation<br>Cell infiltration<br>Tumor response	• DTH to DNP- was induced in 22/24 patients<br>• Inflamed metastatic lesions demonstrated marked lymphocyte infiltration, predominantly CD8+ T cells expressing HLA-DR.<br>• Tumor regression observed in 5 patients; disease stabilization for 3-6 months in 2 patients	[39]
n=12 patients with surgically incurable, metastatic melanoma	**Single-arm treatment group**: DNP-modified autologous tumor vaccine	Autologous, cryopreserved melanoma cells<br>Irradiated (25 Gy)<br>Haptenated<br>Mixed with BCG	Pre-vaccination sensitization using topical 1% DFNB<br>CY (300 mg/m² i.v.) before sensitization and treatment<br>DNP-vaccine injections were administered every 28d	Post-treatment tumor biopsies collected **2–6 months** after therapy initiation (median ≈ 3.5 months)	Immunophenotyping of infiltrations<br>Histopathology	• T-cell infiltration increased significantly in post-treatment tumors (vs. pre-treatment; median 41% vs. 9%, $P < 0.05$)<br>• TILs were predominantly CD8⁺ (median CD8/CD4 ratio = 5.0) with high expression of activation markers: HLA-DR (48%), CD69 (56%), GD3 (68%)<br>• Histopathology analysis revealed lymphocytic infiltration, satelitosis, and areas of tumor necrosis in treated lesions	[40]
284 patients with metastatic melanoma - stage III (n=261) / IV (n=23)	**Single-arm treatment group**: autologous DNP-modified melanoma cell vaccine	Autologous melanoma cells<br>Irradiated (25 Gy)<br>Haptenated<br>Mixed with BCG	5 vaccination schedules (A–E):<br>ID injections; low-dose CY (300 mg/m² i.v.); +/-DNFB sensitization ; different doses	**A:** 8 monthly doses<br>**B/C:** 12 weekly doses<br>**D/E:** 6 weekly doses with boost	DTH response to modified/unmodified tumor cells or PPD (ctrl)<br>RFS and OS<br>Autoimmunity and local reactions	• 57% of patients developed a positive DTH response (≥ 5 mm) to unmodified tumor cells<br>• Patients who developed strong DTH responses had significantly better survival outcomes<br>• Higher proportions of dead tumor cells in the vaccine were associated with stronger DTH responses<br>• Vaccine was well tolerated with no cases of systemic autoimmunity	[41]
297 patients with metastatic melanoma - Stage III (adjuvant, n=214)/IV (metastatic, n=83)	**Single-arm treatment group**: autologous DNP-modified melanoma cell vaccine	Autologous melanoma cells<br>Irradiated (25 Gy)<br>Haptenated<br>Mixed with BCG	5 vaccination schedules (A–E): i.d. injections; low-dose CY (300 mg/m² i.v.); DNFB sensitization in groups A-C; dose ranges	**A:** 8 monthly doses<br>**B/C:** 12 weekly doses<br>**D/E:** 6 weekly doses with boost	DTH<br>RFS and OS<br>Autoimmunity and local reactions	• ~50% developed DTH to **unmodified tumor cells :** strongly predict longer survival<br>• **Stage IV (metastatic melanoma):** 11 clinical objective responses (2 CR, 4 PR, 5 mixed);<br>• **Stage III :** 5-year survival 46% overall ; DTH response (≥10 mm) OS: 38 months vs DTH response <10 mm OS: 16 months<br>• Safety profile was favorable: mostly local and mild; no systemic autoimmunity observed	[43]
214 patients with stage III melanoma<br>Stage IIIC (n=117) / IIIB (n=97)	**Single-arm treatment group**: autologous, DNP-modified melanoma cell vaccine	Autologous melanoma cells<br>Irradiated (25 Gy)<br>Haptenated<br>Mixed with BCG	4 vaccination schedules (A–D): i.d. injections; low-dose CY (300 mg/m² i.v.); dose ranges	**A**: vaccine *before* CY<br>**B-D**: vaccination given *after* CY at varying intervals	OS and RFS<br>Post-relapse survival<br>DTH	• 5-year OS: 44%; 5-year RFS: 33%<br>• Patients with positive DTH (≥ 5 mm) to unmodified tumor cells had significantly higher 5-year OS (59.3% vs. 29.3%)<br>• DTH to unmodified tumor cells is higher after induction doses, at shorter intervals<br>• Vaccine was well tolerated, with only mild local reactions and no cases of systemic autoimmunity	[44]
27 patients with metastatic melanoma	**Single-arm treatment group**: DNP-modified autologous melanoma cell vaccine	Autologous melanoma cells<br>Irradiated (25 Gy)<br>Haptenated	CY (300 mg/m² i.v.) before DNP sensitization and before each vaccination cycle<br>ID administration of treatment - 4 week intervals	Vaccination was continued for **up to 8 months**	DTH<br>*l* PBLproliferation<br>Cytokine production<br>Cytotoxicity assays<br>Anti-DNP antibody responses	• PBL proliferation in response to DNP-modified autologous cells was observed<br>• Patients developed IFN-γ–producing T cells (5/11), including CD8⁺ cytotoxic cells capable of MHC-restricted lysis of DNP-modified melanoma cells.<br>• Anti-DNP antibodies were dosed in patient's serum post treatment.	[45]
43 patients: 28 with AJCC Stage III and 15 with AJCC Stage IV melanoma	**Single-arm treatment group**: adjuvant therapy with a DNP-modified autologous melanoma cell vaccine	Autologous melanoma cells<br>Irradiated (110 Gy)<br>Haptenated<br>Mixed with BCG	CY (300 mg/m²)<br>Topical DNP sensitization<br>8 vaccine doses over ~7 months, every 3–4 weeks	Approx. 7 months	DTH<br>DFS<br>OS<br>Safety and tolerability	• DTH response (≥ 10 mm erythema) correlated with better OS (63 vs. 16 months for DTH-negative)<br>• Multivariate analysis: weak/absent DTH associated with higher risk of recurrence and death.<br>• Vaccine was safe, with no significant systemic toxicity	[46]
34 patients with stage III/IV metastatic malignant melanoma	**Primary treatment**: 27 patients received vaccine for active metastatic disease<br>**Adjuvant setting**: 7 patients vaccinated after resection but later relapsed	Autologous melanoma cells<br>Irradiated (110 Gy)<br>Haptenated<br>Mixed with BCG	CY (300 mg/m²)<br>Topical DNP sensitization<br>8 vaccine doses over ~7 months, every 3–4 weeks		Tumor Response: CR, PR, stable disease, progression<br>DTH<br>OS<br>Safety and tolerability	• Longer median OS when strong DTH compared with those with weak or negative DTH (54 vs. 21 months, $P = 0.013$)<br>• ORR: 35% (4 CR [11.8%], 8 PR [23.5%])<br>• IL-2 enhances vaccine efficacy: 10/12 responders received IL2<br>• Durable responses: The tumor regressions (CR or PR) achieved after IL-2 + vaccine lasted a median of 6 months<br>• No unexpected severe toxicities were reported. Two patients, both in whom tumour regressions occurred developed vitiligo	[47]
47 patients with AJCC stage III/IV melanoma<br>Adjuvant group: 23 patients<br>Therapeutic group: 24 patients	**Adjuvant group**: post-surgical patients<br>**Therapeutic group**: patients with unresectable stage IV active melanoma	Autologous melanoma cells<br>Irradiated (170 Gy)<br>Haptenated<br>Mixed with BCG	CY (300 mg/m²)<br>Topical DNP sensitization<br>Intradermal vaccine doses on D21 and every 3–4 weeks (up to 8 doses)	Core protocol: 8 vaccine doses administered over 5–6 months<br>Boost injections: administered 3, 6, and 12 months	Clinical: OS, DFS, and objective tumor regression<br>Immunologic: $CD4^+/CD8^+$ IFNγ ELISPOT, serum anti-livin and anti-gp100 IgG levels, DTH	• $CD8^+$ T-cell responses increased post-vaccination ($P = 0.035$)<br>• $CD4^+$ T-cell responses correlated with OS in adjuvant group ($P = 0.04$)<br>• Increased serum IgG against livin correlated with **prolonged overall survival ($P = 0.038$)**<br>• MHC II expression on tumor cells was crucial for eliciting post-vaccination $CD4^+$ and $CD8^+$ responses	[50]
[illegible]	[illegible]	Autologous melanoma cells	8 s.c. vaccine doses, every 3 weeks		OS and DFS	• No grade 3–4 adverse events reported	

126 patients with resected AJCC Stage IIIB (n=57) and IIIC (n=69) melanoma	**Single-arm treatment group**: autologous DNP-modified melanoma cell vaccine	Irradiated (230 Gy)<br>Haptenated<br>Mixed with BCG	CY (300 mg/m²)<br>Topical DNP sensitization 1	Treatment lasted ~5 months (21–24 weeks)	DTH<br>CTA gene expression<br>Ipilimumab responses	• 5-year OS: 54% overall; 75% in strong DTH (≥15 mm) responders vs. 44% in weak responders (HR = 0.24; 95% CI 0.10–0.53; $P < 0.001$)<br>• CTA gene expression (CTAG2, MAGEA1, SSX1, SSX4) correlated with better survival<br>• Ipilimumab response: 3-year OS 46% in vaccinated vs. 19% in non-vaccinated, $P = 0.007$	[48]
10 melanoma patients with post-vaccine inflamed metastases	**Single-arm treatment group**: autologous, DNP-modified melanoma cell vaccine	Autologous melanoma cells<br>Irradiated (25 Gy)<br>Haptenated<br>Mixed with BCG	Intradermal injection of DNP-modified autologous melanoma cells mixed with BCG	Not fixed; patients received serial doses of the cell vaccine,	TCR repertoire analysis<br>Tumor infiltration by T-cells<br>Tumor regression and patient survival	• Clonal T-cell expansions were detected in 9 of 10 post-vaccine tumors,<br>• Clonal expansions were tumor-restricted, absent in matched PBL and control tumors<br>• **Inflamed lesions** showed **dense intratumoral CD8⁺ T-cell infiltration**<br>• **Associated with partial regression** of metastases in **3 of 4 evaluable cases**	[28]
**Glioblastoma**							
Interim analysis: 9 patients	**Intervention arm:** ERC1671 + GM-CSF + low-dose CY + bevacizumab (n=5)<br>**Control arm:** Placebo + bevacizumab (n=4)	Autologous + Allogeneic tumor cells and lysates<br>Haptenated<br>Irradiated (25 Gy)<br>Mixed with GM-CSF (500 µg)	28-day cycle:<br>*Day 1:* Bevacizumab (10 mg/kg)<br>*Days 2–5:* CY<br>*Days 6–18 (±1):* 5 vaccine (or placebo) injections + GM-CSF<br>*Day 15:* 2nd bevacizumab dose	Cycles repeated every 28d until disease progression or intolerance<br>MRI scans every 8 weeks for tumor assessment	OS, PFS, and radiologic response<br>CD4⁺ T-cell counts<br>Adverse events graded using CTCAE v4.0	• Median OS: 12.1 months (ERC1671 + bevacizumab) vs. 7.6 months (placebo + bevacizumab)<br>• Radiologic response rate: 75% in ERC1671 arm vs. 25% in placebo arm<br>• CD4⁺ T-cell counts strongly correlated with OS in ERC1671 group only (R = 0.98, $P = 0.02$)<br>• Vaccine was well tolerated, no grade 4/5 toxicities reported (injection site reaction and headache most frequent side effect)	[49]
21 patients with rGBM	**Single-arm treatment**: SITOIGANAP + GM-CSF + low-dose oral CY + bevacizumab + anti-PD-1 (nivolumab or pembrolizumab)	Autologous + Allogeneic tumor cells and lysates from 3 HLA-matched donors<br>Haptenated<br>Irradiated (25 Gy)	28-day cycle:<br>*D1:* Bevacizumab + PD-1 inhibitor (nivolumab or pembrolizumab)<br>*D2-5:* CY<br>*D6, 9, 12, 15, 18* : SITOIGANAP + GM-CSF	Cycles repeated every 28d until disease progression or intolerance	OS and PFS<br>CD3⁺/CD4⁺ T-lymphocyte counts<br>Safety/adverse events	• Patients receiving ≥ 6 cycles of SITOIGANAP: mOS 30.64 months, 1-year survival 90%<br>• Peripheral CD3⁺/CD4⁺ cell counts positively correlated with OS (r = 0.89, $P = 0.0167$)<br>• No treatment-related serious adverse events observed	[51]

Several publications from the Berd group report overlapping patient cohorts, reflecting serial immunologic and clinical analyses within a single clinical development program; therefore, patient numbers across individual studies should not be considered additive
